# On a New Caustic in Malignant Ulcerations of the Face

**Published:** 1854-01

**Authors:** 


### ARTICLE XVIII.

### *On a New Caustic in Malignant Ulcerations of the Face.*

M. E. Cazenave, of Pau, relates, in "L'Union Médicale" for 22d January, two cases of malignant ulceration of the face, in which he has successfully employed a local application, made from sulphuric acid and powdered saffron. The remedy is formed by pouring the acid on the saffron, and applying it in the form of a soft paste. Its corrosive action is immediately manifested on the diseased tissues; the paste dries, and falls off in two or three days, in the form of black crusts, which carry with them the eschar. The application is made several times; the wound assumes a healthy red tint, and cicatrization takes place. In one case, a year has elapsed, in the other two years; and the disease has not returned.

The efficacy of this treatment is evidently dependent on the sulphuric acid; which, we believe, would succeed equally as well if made into a paste with common flour, or any ligneous powder, as with saffron. A paste of sulphuric acid and flour would be worth trying in obstinate cases of phagedemic ulceration.—*Association Med. Jour.*

